# Dendritic Cells in Sepsis: Pathological Alterations and Therapeutic Implications

**DOI:** 10.1155/2017/3591248

**Published:** 2017-09-18

**Authors:** Dong-Dong Wu, Tao Li, Xin-Ying Ji

**Affiliations:** ^1^School of Medical Sciences, College of Medicine, Henan University, Kaifeng, Henan 475004, China; ^2^Institute of Environmental Medicine, Henan University, Kaifeng, Henan 475004, China; ^3^The Affiliated Nanshi Hospital of Henan University, Nanyang, Henan 473000, China

## Abstract

Sepsis is the leading cause of death for critically ill patients in recent years. Dendritic cells (DCs) are important antigen-presenting cells and play a key role in immune response by regulating the innate and adaptive immunity. The number of DCs, the differentiation of monocytes into DCs, and the levels of surface molecules associated with the function of DCs are changed in the development of sepsis. There are many mechanisms involved in the alterations of DCs during sepsis, including the induction of apoptosis, reactive oxygen species generation, activation of the Wnt signaling pathway, epigenetic regulation, and variation in Toll-like receptor-dependent signaling. In this review, we present the classifications of DC subsets and mechanisms involved in the alterations of DCs in sepsis, as well as further discuss the therapeutic strategies targeting DCs in sepsis to improve the aberrant immune response and prolong the life during sepsis progression.

## 1. Introduction

Sepsis has been considered the leading cause of death in critically ill patients, and the incidence of sepsis is increasing worldwide each year [1–3]. Sepsis is now considered a life-threatening organ dysfunction caused by a dysregulated host response to infection [4]. Sepsis is characterized by an excessive production of inflammatory cytokines such as interleukins, tumor necrosis factor, high mobility group box-1, and macrophage migration inhibitory factor, which can lead to multiple organ dysfunction and death [5, 6]. The clinical symptoms of sepsis include tachypnea, hypoperfusion, tachycardia, hypotension, lactic acidosis, and altered body temperature [6–8]. Despite substantial advances in our knowledge regarding the pathogenesis of sepsis and recent progresses in clinical care, efforts to develop and gain regulatory approval for therapeutic agents for the treatment of sepsis remain unsuccessful [3, 9, 10].

Dendritic cells (DCs) are important antigen-presenting cells and play an important role in immune response by linking the innate and adaptive immunity [11, 12]. DCs arise from bone marrow progenitors that could give rise to circulating DC precursors that seed the peripheral tissues as immature cells [13]. DCs reside in virtually all the tissues of our body in a predominantly antigen-capturing state and maintain immunologic tolerance by routinely migrating to the draining lymph nodes and presenting self-antigens to lymphocytes in a tolerogenic manner [14, 15]. The maturation/activation of DCs is followed by many phenotypical and functional changes, promoting their migration to lymph nodes, the secretion of cytokines, and resulting in the activation of T cells [16, 17]. Furthermore, there is increasing evidence that abnormalities in DC homeostasis are implicated in a variety of human diseases, such as infections [18].

In this review, we highlight recent studies that provide new insights into the classifications of DC subsets and mechanisms involved in the alterations of DCs in sepsis, as well as discuss the therapeutic strategies targeting DCs in sepsis to improve the aberrant immune response and prolong the life during sepsis progression.

## 2. DC Subsets

Based on recent classifications of DC subsets, DCs could be divided into two major groups: classical and nonclassical DCs [19]. DC subsets and their basic functions in mice are shown in Table 1.

### 2.1. Classical DCs

Classical or conventional DCs (cDCs) are a highly specialized DC subset that can play an important role in antigen processing and presentation in human organs and tissues. cDCs are involved in the maintenance of immunological homeostasis during the steady state and could be categorized as migratory and lymphoid tissue resident DCs [20]. Lymphoid tissue resident DCs have been found in the thymus, spleen, and lymph nodes [21]. Migratory DCs develop from precursors in both lymphoid and nonlymphoid tissues but are not detected in the spleen [22]. cDCs could be distinguished according to the expression of cell surface markers including cluster of differentiation (CD) 11b, CD8, and CD103 [23, 24]. cDCs can be mainly divided into two subpopulations: CD11b^+^ and CD11b^−^ cells. CD11b^+^ DCs are an interferon regulatory factor 4-dependent subset of lymphoid resident DCs and play a key role in presenting antigens on major histocompatibility complex class II (MHC II) to CD4^+^ T cells [25]. CD11b^−^ DCs include nonlymphoid tissue CD103^+^CD11b^−^ DCs and lymphoid tissue CD8a^+^CD11b^−^ DCs [23]. CD8^+^ cDCs play a vital role in immune responses against many different types of viruses and intracellular bacteria. CD103^+^ cDCs populate nonlymphoid tissues where they regulate immune tolerance to food antigens and commensal bacteria [24, 26]. The CD8^+^ and CD103^+^ cDC lineage development is controlled by many transcription factors, including inhibitor of DNA binding 2, basic leucine zipper transcriptional factor ATF-like 3, nuclear factor interleukin-3-regulated protein, and interferon regulatory factor 8 [27–30].

### 2.2. Nonclassical DCs

Nonclassical DCs can be further divided into three major subsets: plasmacytoid DCs (pDCs), monocyte-derived DCs (moDCs), and Langerhans cells (LCs) [24]. pDCs arise from lymphoid progenitors and are broadly distributed throughout the body. The DC subset can be identified through the expression of CD45R and immunoglobulin-like transcript 7 in humans [20, 31]. pDCs are efficient antigen-presenting cells specialized in the production and secretion of type I interferons (IFNs) following their recognition of viruses or self-nucleic acids through Toll-like receptor (TLR) 7 and TLR9 [23, 32]. pDCs also produce other proinflammatory cytokines/chemokines, such as interleukin- (IL-) 6, IL-12, CC-chemokine ligand 3 (CCL3), CCL4, CXC-chemokine ligand 8 (CXCL8), and CXCL10, which recruit immune cells to sites of inflammation or infection [32]. moDCs (also known as inflammatory DCs), originating from monocytes, are induced by infection, inflammation, or cancer, and they do not accumulate under steady-state conditions [23, 33]. moDCs play a crucial role in immune responses because they provide a number of antigen-presenting cells that can effectively initiate an adaptive immune response following the onset of infection [20]. Sufficient numbers of autologous moDCs can be easily obtained from peripheral blood of patients [34]. LCs originate prenatally and could endure throughout life, independently of bone marrow-derived precursors [35]. LCs reside in the epidermal skin layer and can be identified by the lymphocyte antigen 6C [36]. After their maturation by pathogen-related molecules, LCs migrate to lymph nodes via the afferent lymphatics and present antigens to activate naive T cells [24, 37].

## 3. Alterations of DCs in Sepsis

**Figure 1:**
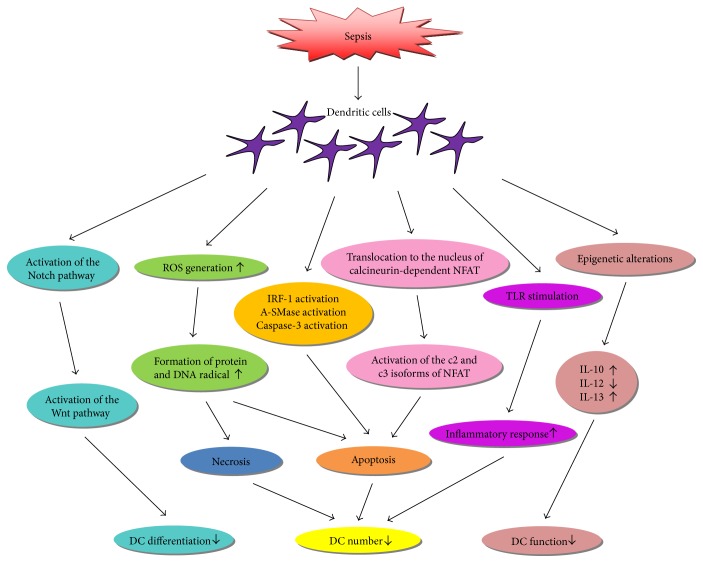
A schematic illustration of the alterations of dendritic cells (DCs) in sepsis. Induction of apoptosis, reactive oxygen species (ROS) generation, activation of Wnt signaling pathway, epigenetic regulation, and variation in toll-like receptor- (TLR-) dependent signaling are involved in the alterations of DCs in sepsis. NFAT: nuclear factor of activated T cells; IRF1: interferon regulatory factor 1; A-SMase: acid sphingomyelinase; DNA: deoxyribose nucleic acid; IL: interleukin.

During sepsis, the number of DCs is decreased but the differentiation of monocytes into DCs is accelerated [38–42]. The levels of surface molecules associated with the function of DCs in sepsis are altered [43]. Dysfunctional DCs cocultured with T cells could lead to T cell anergy [44]. In addition, septic DCs show an aberrant cytokine secretion which results in immune tolerance status [12, 43, 45, 46]. Recent findings indicate that there are several mechanisms contributing to the alterations of DCs in sepsis, including induction of apoptosis, activation of the Wnt signaling pathway, reactive oxygen species (ROS) generation, variation in TLR-dependent signaling, and epigenetic regulation (Figure 1).

### 3.1. Induction of Apoptosis

Apoptosis, also known as programmed cell death, is an intrinsic cell-suicide program that plays a key role in the maintenance of tissue homeostasis and normal development in multicellular organisms [47, 48]. It has been observed that DC apoptosis contributes to the development of immunosuppressive state and organ injury during sepsis [43, 49, 50]. Caspase-3-mediated apoptosis of DCs results in immunosuppression, which can be observed both in humans and in mouse models of sepsis, and suppression of DC apoptosis in mice leads to resistance to endotoxin-induced sepsis [50–52]. In addition, a recent study has shown that overexpression of B-cell lymphoma 2 (Bcl-2) could dispel sepsis-induced depletion of DCs, suggesting that the proteins involved in apoptosis play an important role in DC loss during sepsis [53]. The lipopolysaccharide (LPS) injection/infusion model has been widely used for sepsis research [54]. The mechanism of DC apoptosis induced by LPS requires the activation of c2 and c3 isoforms of nuclear factor of activated T cells [55]. Many studies have shown that a mammalian TLR-dependent pathway is also involved in the process of sepsis-induced DC apoptosis [38, 56]. Furthermore, another study indicates that the apoptosis of immature DCs induced by high concentrations of LPS requires the activation of acid sphingomyelinase [57]. Currently, the mechanisms of DC apoptosis induced by sepsis have not been fully elucidated; more efforts should be made to clarify the underlining mechanisms. Inhibition of DC apoptosis may be a novel therapeutic target for sepsis.

### 3.2. Activation of Wnt Signaling Pathway

The Wnt signaling pathway includes a large family of highly conserved proteins that are required for basic developmental processes [58]. Wnt proteins have been considered endocrine factors involved in several diseases, such as septicemia and cancer [59, 60]. Many studies have indicated that DCs are important targets for the immunomodulatory activity of Wnt signaling [61]. Wnt5a is a noncanonical Wnt protein that is involved in cell migration, adhesion, and tissue polarity [62]. A recent study shows that Wnt5a-induced IL-6 and concurrent inhibition of extracellular signal-regulated protein kinase 1/2 activity could inhibit the differentiation of monocyte-derived myeloid dendritic cells, suggesting that Wnt5a may act as a candidate mediator for the CD14^+/++^CD16^+^ monocyte accumulation in patients with sepsis [63]. Another study indicates that Wnt5a could be an important factor that contributes to the dysfunction of DCs that develops during polymicrobial sepsis [46]. In addition, the activation of the Wnt canonical pathway by Wnt3a could promote the degeneration of CD11c^+^ DCs and stimulate T cell proliferation [64]. Therefore, the identification of Wnt proteins may improve the design of more effective immunotherapeutic strategies for the treatment of infection and sepsis [61].

### 3.3. ROS Generation

ROS are defined as partially reduced metabolites of molecular oxygen that possess strong oxidizing capabilities [65]. The most important and widely studied members of ROS are the superoxide anion, hydroxyl radical, and hydrogen peroxide [66]. ROS have been considered as cellular signaling molecules and mediators of inflammation. Production of ROS plays a key role in the progression of many inflammatory diseases [65]. It has been reported that increased ROS generation coupled with deoxyribose nucleic acid (DNA) and protein radical adduct formation could result in rapid depletion of follicular DCs from the septic spleen [67]. Another study indicates that LPS-induced ROS generation and the concomitant decline in both reduced glutathione and oxidized glutathione are likely involved in the maturation of human moDCs [68]. In conclusion, the regulation of ROS generation could be a useful therapeutic tool in diseases in which immune and inflammatory responses become entangled, such as sepsis.

### 3.4. Variation in TLR-Dependent Signaling

TLRs are important components of the innate immune system that detect microbial infection and induce antimicrobial host responses [69]. TLR family members can be detected in many subcellular compartments, such as the plasma membrane and early, late, and recycling endosomes [70]. Interaction of TLRs with their ligands results in the activation of downstream signaling pathways that trigger an immune response by producing type I interferons, inflammatory cytokines, and other inflammatory mediators [71]. Variability in the activation of TLR-dependent signaling pathways is involved in regulating the magnitude of the innate immune response and the efficiency of host defense mechanisms [72]. Although dispensable for the process of DC maturation, TLR2 and TLR4 play key roles in the mechanisms resulting in the depletion of spleen DC following polymicrobial sepsis [38]. It has been found that the absence of TLR9 signaling promotes the local influx of DCs during peritoneal sepsis, which is associated with an enhanced granulocyte response that is necessary for survival [73]. These findings indicate that the detrimental immune response to bacterial sepsis occurs via the variation in TLR-dependent signaling. Targeting the TLR-dependent signaling could be a potential strategy for the treatment of sepsis.

### 3.5. Epigenetic Regulation

Epigenetic regulation refers to reversible and heritable changes in gene expression without affecting DNA sequences [74]. The main epigenetic mechanisms include DNA methylation, histone modifications, and regulation by noncoding ribose nucleic acids [75]. Many studies have shown that epigenetic mechanisms play vital roles in embryogenesis, inflammation, and cancer [76–78]. Recently, an increasing amount of evidence suggests that epigenetic mechanisms, driven by unknown signals generated during the process of sepsis, are involved in mediating postseptic immunoaberrancy [79]. A potential epigenetic-dependent mechanism involved in alterations in DC number and function has been proposed as the underlying etiology of long-term postseptic immunosuppression [80, 81]. Another study provides evidence for the changes in histone methylation and characterizes many histone methyltransferase complexes associated with the regulation of DC-derived IL-12 in postseptic animals. These epigenetic changes play important roles in facilitating stable alterations in cytokine gene expression, which mechanistically contribute to the long-term immunosuppression after severe sepsis [82]. More evidence of the epigenetic regulation in DC dysfunction after sepsis will be a benefit for the development of novel cell and mediator-based therapeutic interventions.

## 4. Therapeutic Strategies Targeting DCs in Sepsis

Considering the pivotal role of DCs in the immune activation and survival in sepsis, the modification of DC system during sepsis is becoming an increasingly important area of investigation [51, 73, 83]. To date, a number of strategies have been developed and successfully used to improve the aberrant immune response and prolong the life during sepsis progression.

### 4.1. Improvement of DC Survival

Recent studies have indicated that profound depletion of DC is a specific hallmark in both septic patients and experimental animal models of sepsis [38, 39, 84]. *DC-hBcl-2* mice are a transgenic mouse model specifically overexpressing Bcl-2 in DCs. The DCs derived from *DC-hBcl-2* mice exhibit higher resistance to maturation-induced apoptosis after LPS treatment. Additionally, prolongation of DC survival decreases sublethal LPS-induced DC loss and immunosuppression, with enhanced T cell activation and maintenance of the differentiation potential of Th1 cells. This study indicates that DC death is a key determinant of endotoxin-induced immunosuppression and mortality in mice and modulation of the immune response may play an important role in attenuating mortality observed after LPS-induced shock [51]. TLR9 is involved in the activation of innate immunity against microbial pathogens. Compared with wild-type (WT) mice, TLR9^−/−^ mice have shown higher bacterial clearance, lower serum levels of inflammatory cytokines, and longer survival after experimental peritonitis induced by cecal ligation and puncture (CLP). Protection of TLR9^−/−^ mice after CLP can be attributed to a greater number of peritoneal DCs and granulocytes than those in WT mice, suggesting that TLR9 blockade may be a useful strategy for the treatment of human sepsis [73]. Human telomerase reverse transcriptase (hTERT) has been widely considered a catalytic enzyme required for telomere elongation [85]. A recent study has shown that the median survival time of DCs transfected with hTERT is significantly higher than that of the untransfected DCs in a LPS-induced sepsis mouse model. In addition, the hTERT transfecting DCs could reduce apoptosis and cytokine secretion, as well as decrease the inflammatory response in septic mice [86]. Therefore, hTERT could be a promising molecular target in preventing the progression of sepsis and increasing the survival time.

### 4.2. Modification of DC Function

Considering the crucial roles in the innate and acquired immune responses, modification of DC function may be a promising way for the development of cellular therapeutics for cancer and immune-mediated processes [87]. IL-10, a pleiotropic cytokine, plays an important role in regulating the development and function of numerous cells [88–90]. DCs have been shown to be permissive to adenovirus (Adv) infection at high particle concentrations [91]. It has been shown that DCs transduced with Adv/IL-10 maintain an immature state with low expressions of IL-12, CD86, and MHC II [87]. Furthermore, Adv/IL-10 transduction of DCs significantly improves the survival of septic mice, indicating that compartmental modification of DC function alters the sepsis-induced immune response [87, 92]. Glucocorticoids (GCs) are steroid hormones that play key roles in a variety of essential cardiovascular, metabolic, and homeostatic functions [93–95]. IL-12 is essential for IFN-*γ* production and lethality in LPS-induced septic shock. Elevation of GCs that accompanies sepsis protects mice from LPS-induced septic shock through the suppression of DC-derived IL-12, a cytokine that can cause the secretion of other inflammatory mediators [84, 96]. Janus kinase 2 (Jak2) is crucial for the regulation of DC function and development [97, 98]. Deficiency of Jak2 selectively inhibits DC-mediated innate immune response and protects mice from LPS-induced septic shock [99], suggesting that blocking the IFN-receptor signaling may avoid a deleterious immune response. It has been shown that TLR4 antagonist could inhibit LPS-induced cytokine production and glycolytic reprogramming in DCs [100]. Eritoran, an antagonist of TLR4, exhibits positive results in phase I and phase II clinical trials of severe sepsis, but it has failed in a phase III-randomized controlled trial [101]. The treatment of severe sepsis with TLR4 antagonist may be limited to selected patients. In addition, a recent study has indicated that CD155 blockade could improve survival by reversing DC dysfunction in experimental sepsis [102]. These results indicate that IL-10, IL-12, Jak2, TLR4, and CD155 could be promising therapeutic targets for the intervention and treatment of clinical septic shock.

### 4.3. Alteration of DC Distribution

DCs are important antigen-presenting cells that are involved in the regulation of innate and adaptive immune responses [103–105]. The exposition to an immunological stimulus results in DC migration into regional lymph nodes where antigen presentation to naive T cells takes place. During migration, DCs undergo several phenotypic and functional alterations, characterized by the upregulation of MHC II and costimulatory molecules [106–108]. C5a, a potent chemoattractant, is excessively activated during the onset of sepsis [109]. C5a exhibits many biological functions including modulation of cytokines expression and regulation of adaptive immune responders in particular regulatory T cells [110, 111]. C5a could induce IL-12^+^DC cell migration from the peritoneal cavity to lymph nodes and peripheral blood where IL-12^+^DC cells induce the expansion of pathogenic IL-17^+^T helper (Th) 17 and IFN*γ*^+^Th1 cells. IL-12, secreted by DC cells in the peritoneal cavity, has a protective effect in preventing the development of sepsis [109]. However, the role of IL-12 in LPS-induced lethality is controversial [84]. More efforts are needed to illuminate the underlying mechanism of action of IL-12 in the process of sepsis.

## 5. Discussion

It is widely accepted that DCs are important antigen-presenting cells involved in immune response by linking the innate and adaptive immunity. DCs mainly include two groups: classical and nonclassical DCs. Nonclassical DCs can be further divided into three subsets, including LCs, pDCs, and moDCs. Whether there exists another subset of DCs should be further studied and confirmed. It has been shown that the number of DCs, the differentiation of monocytes into DCs, and the levels of surface molecules associated with the function of DCs are changed in sepsis. Furthermore, septic DCs show an aberrant cytokine secretion which results in immune tolerance status. An increasing number of studies suggest that there are many mechanisms involved in the alterations of DCs in sepsis, such as induction of apoptosis, ROS generation, activation of the Wnt signaling pathway, epigenetic regulation, and variation in TLR-dependent signaling. Novel mechanisms associated with the alterations of DCs in sepsis need to be further studied and illuminated, which will inevitably contribute to the development of novel antisepsis drugs.

In light of the key roles of DCs in the immune activation and survival in sepsis, the modifications of DCs during sepsis have become an increasingly important area of research. Recently, many strategies have been developed and successfully used to improve the aberrant immune response and prolong the life during sepsis progression, including the improvement of DC survival, modification of DC function, and alteration of DC distribution. The reduction of the level of autophagy in DCs could be a novel effective strategy in preventing the process of sepsis. More specific biomarkers need to be discovered and applied in the regulation of the function and development of DCs. Furthermore, novel phenotypic and functional modifications of DCs in sepsis should be designed and adopted in fighting against sepsis. Moreover, blocking the biomarkers and/or signaling pathways in DCs could be new therapeutic approaches in reducing lethality in sepsis. Although there is substantial research in mouse models of sepsis, few of these promising findings have been shown to be effective in septic patients, which may attribute to the differences between human and mouse DCs in some of their phenotypes and/or functional properties [112, 113]. Therefore, the novel animal models that most closely resemble the course of sepsis observed in patients should be established and more in-depth research in human sepsis should be conducted.

In conclusion, with a deeper understanding of the precise molecular mechanisms involved in the alterations of DCs in sepsis, novel therapeutic strategies targeting DCs in sepsis could be promising strategies in preventing the development and progression of sepsis.

## Figures and Tables

**Table 1:** DC subsets and their basic functions in mice.

Subsets	Sites	Transcriptional factors	Phenotypic markers	TLR expressions	Functions
cDC	Lymphoid organ	Irf4, Rbpj, Batf3, Irf8	CD11b, CD11c, CD172a, CD103, CD8*α*, XCR1, Clec9a	TLR1, 2, 3, 4, 6, 7, 8, 9	Antigen presentation, induction of antitumor responses, induction of Th2 T cell responses, migration, antigen cross presentation
pDC	Most organs	Tcf-4	CD11c, B220, Ly6C, Siglec-H	TLR1, 2, 4, 5, 6, 7, 8, 9	Antigen presentation, type I IFN production, tumor killing
moDC	Most organs	Unknown	CD11b, F4/80, Ly6c, CD206, CD64, MHC II, Mac-3/CD107b Fc*ε*RI, CD115/GM-CSFR	TLR2, 3, 4, 7, 9	Antigen presentation, induction of antitumor responses, migration, production of TNF and NO, tumor rejection
LC	Skin	PU.1, ID2, Irf4, Irf8	CD36, CD1a, CD1c, CD207, HLA-DR, CD86	TLR1,3,6,7	Antigen presentation, radioresistance, adaptive immunity

cDC: classical DC; Irf4: interferon regulatory factor 4; Rbpj: recombination signal binding protein-J; Batf3: basic leucine zipper transcriptional factor ATF-like 3; IFN: interferon; Irf8: IFN regulatory factor 8; CD: cluster of differentiation; XCR1: X-C motif chemokine receptor 1; Clec9a: C-type lectin domain family 9, member A; TLR: Toll-like receptor; Th2: T helper type 2; pDC: plasmacytoid DC; Tcf-4: transcription factor 4; Siglec-H: sialic acid-binding immunoglobulin-like lectin-h; moDC: monocyte-derived DC; MHC II: major histocompatibility complex class II; Fc*ε*RI: high-affinity immunoglobulin E receptor; GM-CSFR: granulocyte-macrophage colony-stimulating factor receptor; TNF: tumor necrosis factor; NO: nitric oxide; LC: Langerhans cell; ID2: inhibitor of DNA binding 2; HLA-DR: human leukocyte antigen DR.
